# Impact of Arterial Remodeling of Intermediate Coronary Lesions on Long-Term Clinical Outcomes in Patients with Stable Coronary Artery Disease: An Intravascular Ultrasound Study

**DOI:** 10.1155/2021/9915759

**Published:** 2021-06-11

**Authors:** Liang Geng, Peizhao Du, Yuan Yuan, Liming Gao, Yunkai Wang, Jiming Li, Qi Zhang

**Affiliations:** ^1^Department of Cardiology, Shanghai East Hospital, School of Medicine, Tongji University, Shanghai 200092, China; ^2^Department of Cardiology, Baoshan Hospital of Integrated Traditional Chinese and Western Medicine, Shanghai 201900, China

## Abstract

**Background:**

Treatment of coronary intermediate lesions remains a controversy, and the role of arterial remodeling patterns determined by intravascular ultrasound in intermediate lesion is still not well known. The aim of this study was to investigate the impact of arterial remodeling of intermediate coronary lesions on long-term clinical outcomes.

**Methods:**

Arterial remodeling patterns were assessed in 212 deferred intermediate lesions from 162 patients after IVUS examination. Negative, intermediate, and positive remodeling was defined as a remodeling index of <0.88, 0.88∼1.0, and >1.0, respectively. The primary endpoint was the composite vessel-oriented clinical events, defined as the composition of target vessel-related cardiac death, target vessel-related myocardial infarction, and target vessel revascularization. Quantitative flow ratio was assessed for evaluating the functional significance of intermediate lesions.

**Results:**

72 intermediate remodeling lesions were present in 66 patients, whereas 77 negative remodeling lesions were present in 71 patients, and 63 positive remodeling lesions were present in 55 patients. Negative remodeling lesions had the smallest minimum lumen area (4.16 ± 1.03 mm^2^ vs. 5.05 ± 1.39 mm^2^ vs. 4.85 ± 1.76 mm^2^; *P* < 0.01), smallest plaque burden (63.45 ± 6.13% vs. 66.12 ± 6.82% vs. 71.17 ± 6.45%; *P* < 0.01), and highest area stenosis rate (59.32% ± 10.15% vs. 54.61% ± 9.09% vs. 51.67% ± 12.96%; *P* < 0.01). No significant difference was found in terms of quantitative flow ratio among three groups. At 5 years follow-up, negative remodeling lesions had a higher rate of composite vessel-oriented clinical event (14.3%), compared to intermediate (1.4%, *P*=0.004) or positive remodeling lesions (4.8%, *P*=0.06). After adjusting for multiple covariates, negative remodeling remained an independent determinant for vessel-oriented clinical event (HR: 4.849, 95% CI 1.542–15.251, *P*=0.007).

**Conclusion:**

IVUS-derived negative remodeling is associated with adverse long-term clinical outcome in stable patients with intermediate coronary artery stenosis.

## 1. Introduction

Risk stratification and management strategy of intermediate coronary lesions, defined as 50–70% diameter stenosis (DS) at coronary angiography [1, 2], remain a challenging issue [3, 4]. As a rule, percutaneous coronary intervention (PCI) with drug-eluting stent implantation for every intermediate lesion could increase the risk of stent thrombosis or restenosis, whereas deferral revascularization of high-risk intermediate lesions might be associated with a higher risk of long-term events [5, 6]. Intravascular ultrasound (IVUS) is superior to coronary angiography in terms of accurate assessment of lumen area and plaque burden and thus is commonly applied to evaluate intermediate stenotic lesions and guide the decision about revascularization in real-world practice [4, 7]. However, data regarding the long-term clinical outcomes of IVUS-guided deferral of coronary revascularization are limited. Furthermore, previous studies have shown that arterial remodeling assessed by IVUS in vivo affects hemodynamic stress on the lesion site [8–10] and is correlated with clinical presentation [11, 12]. Accordingly, we sought to investigate the impact of arterial remodeling of intermediate lesions on long-term prognosis in patients with stable coronary artery disease.

## 2. Methods

**Figure 1:**
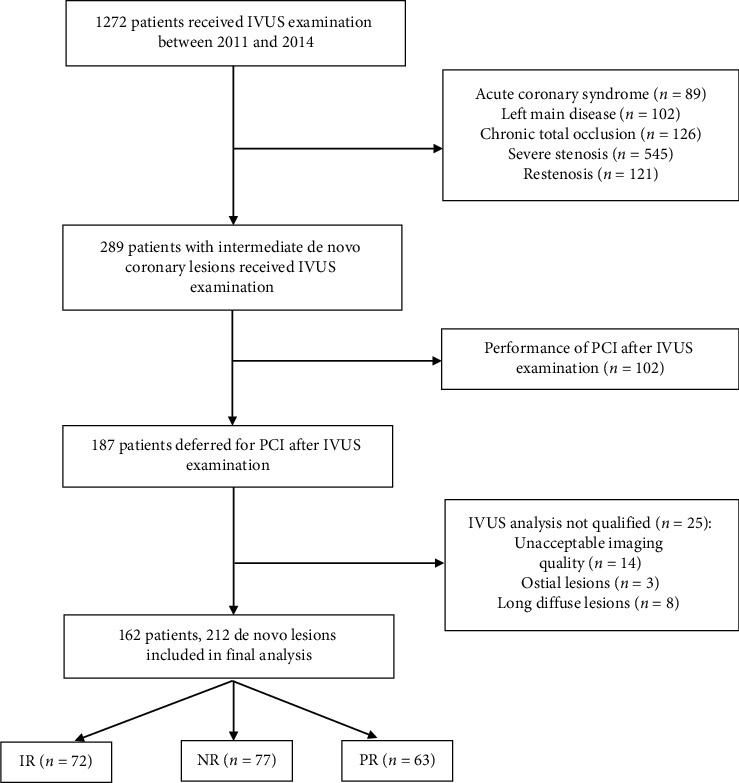
Flowchart of patient enrollment. IR, intermediate remodeling; NR, negative remodeling; PR, positive remodeling.

Consecutive patients with stable coronary heart disease who received IVUS examination between January 2011 and January 2014 were retrospectively screened. Patients were enrolled if they had de novo lesions with a visual estimation of lumen DS of 50–70% and without revascularization (balloon angioplasty and/or stent implantation) after IVUS examination. In our center, IVUS was routinely used to assess the angiographically intermediated lesions. The IVUS criteria for deferral of PCI was minimum lumen area >3.0 mm^2^ or plaque burden <70%. Other clinical scenarios requiring IVUS examination (restenosis, acute coronary syndrome, left main disease, and chronic total occlusion) were excluded. We also excluded patients with ostial lesions (<3 mm from the aorta), long diffuse lesions (>30 mm), and poor imaging qualities. Overall, 162 patients with 212 de novo intermediate coronary lesions were included in the final analysis and divided into three groups according to the remodeling index (Figure 1). Clinical outcomes at five-year follow-up were compared among three groups. The study was approved by the ethical standards of the institutional review board on human experimentation at Shanghai East Hospital, and individual consent for this retrospective analysis was waived.

**Figure 2:**
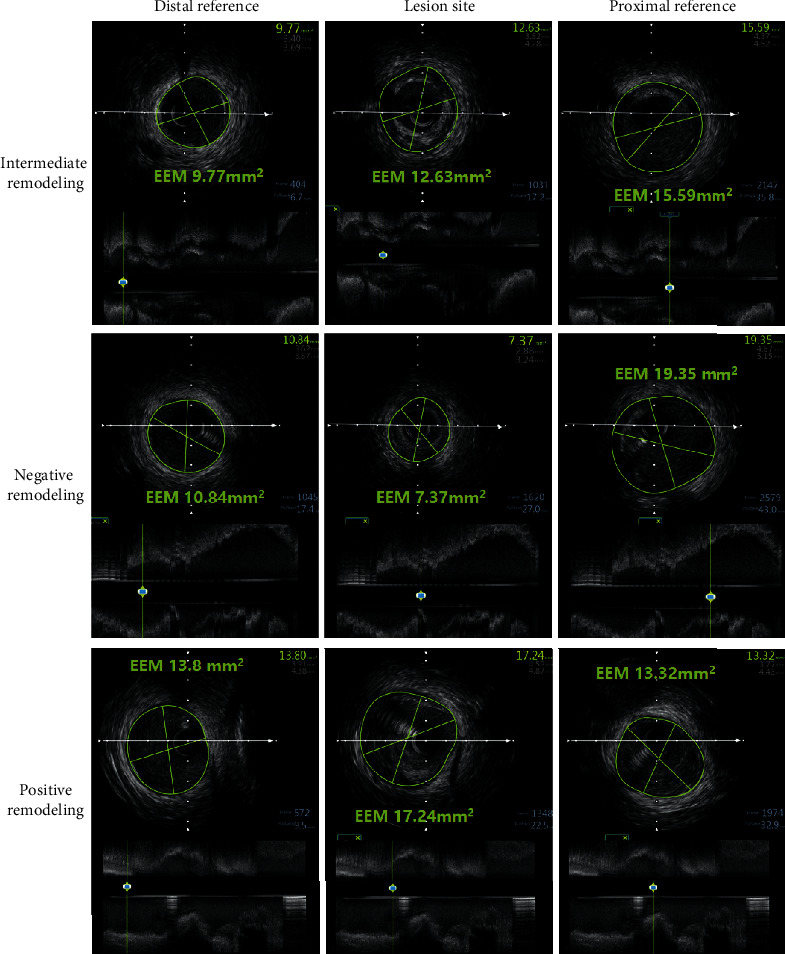
Illustration of arterial remodeling patterns of intermediated lesions.

IVUS examination of intermediate lesions was performed after intracoronary administration of 200 *μ*g of nitroglycerin using the iLab system (Boston Scientific, Natick, Massachusetts, USA). A 40 MHz ultrasound catheter was inserted into the target vessel; then, the transducer was advanced 10 mm distal to the lesion and was automatically pulled back at 0.5 mm/s to aorto-ostial junction. All images were recorded for subsequent analysis. IVUS imaging analysis was made using a commercially available planimetry software (iReview; Boston Scientific, Natick, Massachusetts, USA) by two experienced interventionalists who are blinded to patients' clinical information. The morphological measurements were obtained according to the American College of Cardiology Clinical Expert Consensus Document on Standards for Acquisition, Measurement, and Reporting of Intravascular Ultrasound Studies [13]. Quantitative parameters were assessed at the lesion site and 5 mm proximal and distal reference segments, including lesion length, external elastic membrane (EEM) area, lumen cross-sectional area (CSA), and plaque plus media (P + M) area. The lesion site was defined as the site with the smallest lumen CSA. The definition of proximal and distal reference segment was the site with the largest lumen and smallest plaque burden without any intervening side branch. Lesion length was defined as the distance between proximal and distal reference sites, and area stenosis was calculated as (mean reference lumen CSA-minimum lumen area)/mean reference lumen CSA. Plaque burden (%) was derived from following formula: plaque burden = (P + M) CSA/EEM CSA. Echo-attenuated plaque was defined as the loss of ultrasound signal behind plaque that was in the absence of calcification. Plaque rupture was defined as the presence of a cavity that communicated with the lumen. Plaque was also classified as fibrous, soft, calcified, or mixed one [13]. Remodeling index (RI) was calculated as lesion EEM CSA/average of the proximal and distal reference segment EEM CSAs. Plaque cross-sectional eccentricity was expressed as (maximum−minimum plaque thickness)/maximum plaque thickness. RI > 1.0, 0.88–1.0, and <0.88 were considered as positive, intermediate, and negative remodeling, respectively, according to previous report [14]. The illustration of arterial remodeling patterns is given in Figure 2.

In order to study the potential hemodynamic impact of arterial remodeling, quantitative flow ratio (QFR) of IVUS-guided deferred lesions was calculated by two well-trained investigators using a commercially available QFR system (Angioplus, Pulse Medical Imaging Technology, China). Detailed process of QFR analysis was previously described [15]. Briefly, two angiographic image runs were acquired at different angles (≥25°), and the lumen contour was automatically delineated by an extensively well-validated algorithm to establish the 3D QCA model. Manual correction was only allowed in case of branch or suboptimal image quality. The contrast flow model, which uses a frame count method to derive contrast flow velocity from coronary angiography, was used for final QFR computation in the current study. QFR was analyzed from the ostium of the main vessel to the target vessel where IVUS was interrogated. The distal endpoint was placed at the most distal side branch.

The primary endpoint was the composite vessel-oriented clinical events (VOCE), defined as the composition of target vessel related cardiac death, target vessel related myocardial infarction (MI), and target vessel revascularization (TVR). The secondary endpoints were individual components of the primary endpoint. Cardiac death was defined as any death due to cardiac causes (e.g., MI, heart failure, and fatal arrhythmia). MI was defined as elevation of cardiac troponin (cTn) value with at least 1 value above 99^th^ percentile upper reference limit (URL), with clinical symptoms or ischemic changes or new *Q* waves in at least two contiguous leads on electrocardiogram (ECG) [16]. TVR was defined as any unplanned revascularization of the deferred lesions.

After discharge from index hospitalization, all patients received guideline recommended medications, including at least of aspirin, beta-blocker, and statins [17]. Other medications including angiotensin-converting enzyme inhibitors (ACEI) or angiotensin-receptor blockers (ARB) and calcium channel blockers were prescribed based on individual risk factors. Follow-up was accomplished by outpatient clinic interview or telephone contact at every six months until 5-year. Adverse events were recorded for subsequent analysis. All events were adjudicated by two independent investigators who were blinded to the IVUS data.

Continuous variables are expressed as mean ± standard deviation and compared using Student's *t*-test or one-way ANOVA. Categorical variables are expressed as frequencies (percentages) and compared using the chi-square test. Time to event data was presented as Kaplan–Meier estimates and compared with the log-rank test. The Cox proportional hazard model was used to calculate hazard ratio (HR) and 95% confidence intervals (95% CI) for the between-group comparisons of clinical outcome. The multivariable adjustment was performed after backward selection of clinical risk factors and IVUS indices, with significance for addition to the model set at *P* ≤ 0.15. Variables with significance levels of *P* < 0.1 in univariable analysis and clinically relevant variables were considered for multivariable logistic regression. A 2-sided *P* value <0.05 was considered statistically significant. All statistical analyses were performed with SPSS software, version 22.0 (SPSS institute, Chicago, Illinois).

## 3. Results

Male gender was higher in proportion, and serum levels of creatinine and HbA1C were more elevated in patients with positive arterial remodeling, but the three remodeling patterns did not differ significantly with respect to other traditional risk factors, clinical presentations, and medications at discharge (Table 1).

**Figure 3:**
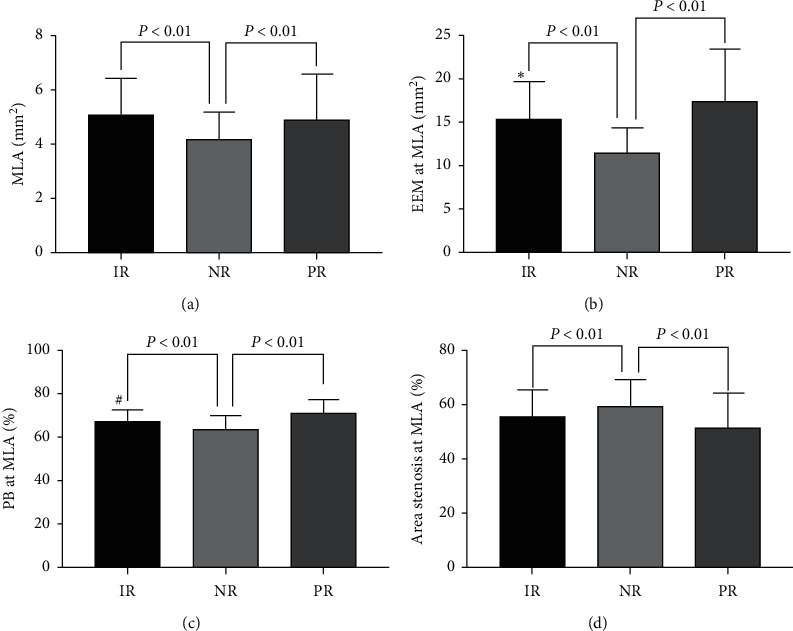
Morphological and quantitative IVUS measurements among lesions with different remodeling patterns. ^*∗*^*P* < 0.01 vs. PR; ^#^*P* < 0.01 vs. PR. EEM, external elastic membrane; IR, intermediate remodeling; MLA, minimum lumen area; NR, negative remodeling; PB, plaque burden; PR, positive remodeling.

The morphological and quantitative results for different remodeling patterns are displayed in Table 2 and Figure 3. Minimum lumen area (MLA) was significantly smaller in negative remodeling lesions compared to those with intermediate or positive remodeling. At the site of MLA, EEM CSA increased, and area stenosis decreased stepwise across negative, intermediate, and positive arterial remodeling groups, leading to a progressive increase in plaque burden from negative remodeling to positive remodeling. Furthermore, soft and echo-attenuated plaques were most common in positive remodeling lesions, whereas fibrous plaques were most prevalent in intermediate remodeling lesions. The incidence of plaque rupture was low and not significantly different among the three groups.

**Figure 4:**
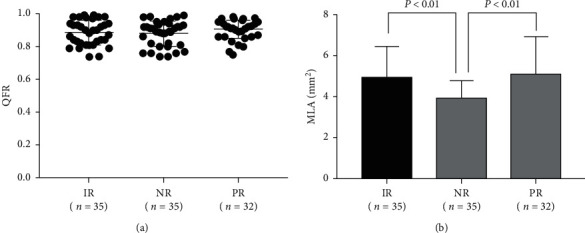
QFR and MLA measurements among QFR analyzable lesions with different remodeling patterns. IR, intermediate remodeling; MLA, minimum lumen area; NR, negative remodeling; PR, positive remodeling; QFR, quantitative flow ratio.

QFR was analyzable in 102 lesions (48.1%) because of lack of autocalibration data (*n* = 55) or appropriated projections (*n* = 32) and poor image quality (*n* = 11) and frame count failure (*n* = 12). Majority of QFR of deferred lesions was above 0.8 (*n* = 88, 86.3%). The mean QFR was not different among the three groups (0.86 vs. 0.87 vs. 0.89, *P*=0.33), whereas the association between smallest MLA and negative remodeling lesions remained viable in the QFR analyzable lesions (Figure 4).

**Figure 5:**
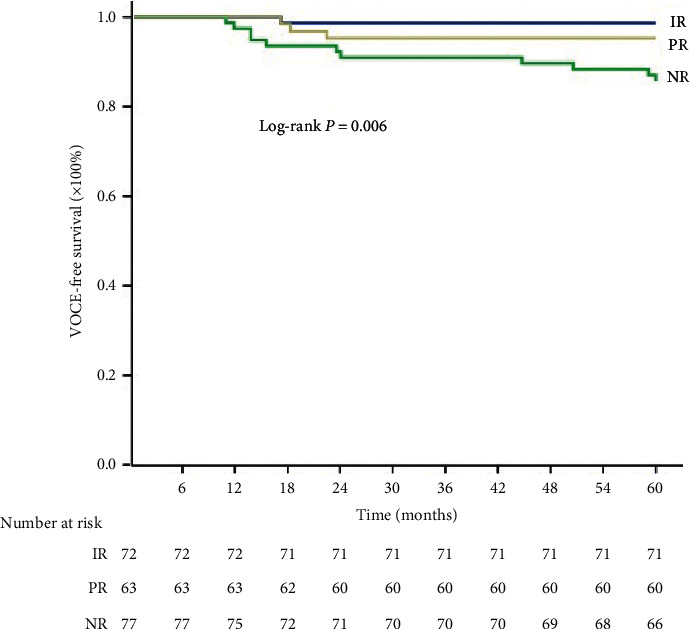
Kaplan–Meier curves for VOCE. IR, intermediate remodeling; NR, negative remodeling; PR, positive remodeling; VOCE, vessel-oriented clinical events.

Clinical follow-ups were available for all patients at 5-year. Overall, VOCEs occurred in 15 lesions (7.1%). Negative remodeling lesions had a higher rate of VOCE (14.3%) compared to those with intermediate (1.4%, *P*=0.004 vs. negative remodeling) or positive remodeling (4.8%, *P*=0.06 vs. negative remodeling). Notably, an increased rate of VOCE was mainly driven by TVR (Table 3). Kaplan–Meier curve analysis showed a higher cumulative incidence of VOCE in the negative remodeling group (*P*=0.006, Figure 5).

After adjustment for potential confounding factors including age, sex, risk factors for coronary artery disease, previous PCI, and multivessel disease, RI <0.88 remained an independent determinant for VOCE (HR 4.849, 95% CI 1.542–15.251, *P*=0.007). Previous PCI was also correlated with VOCE (HR: 3.309; 1.128–9.703; *P*=0.029) (Table 4).

## 4. Discussion

The present study focused on the vascular morphological determinants of long-term outcome of the deferred intermediate lesions after IVUS examination. The main findings are the following: (1) deferral of intermediate lesion revascularization after IVUS examination results in a 5-year VOCE rate of 7.1%, which was mainly driven by TVR; (2) negative arterial remodeling of intermediate coronary lesions (RI <  0.88) was associated with smaller minimum lumen area and higher area stenosis and might predict future worse clinical outcome of deferred lesions; and (3) QFR value did not differ among three remodeling patterns.

Over the years, IVUS is often used for the assessment of severity of coronary artery stenosis in real-world practice and has certain clinical value for risk stratification of intermediate coronary lesions [18, 19]. Previous studies suggested that minimum lumen area <4.0 mm^2^, plaque burden >70%, and IVUS-derived thin-cap fibroatheroma (TCFA) may be predictive for future worse outcomes [20–22]. However, the positive predictive power of these indices remains relatively low [23, 24]. Arterial remodeling appears to be a common adaptive response to hemodynamic stress or arterial injury [25]. Intracoronary imaging studies have demonstrated an existence of the bidirectional remodeling process in the coronary artery [26, 27]. Nevertheless, arterial remodeling at lesion site has long been overlooked, as atherosclerotic plaques have always been considered as the only pivotal determinant of coronary lumen narrowing and patient prognosis. Our study supports a notion that the remodeling pattern of intermediate coronary lesions is an additional important risk factor for the deferred intermediate lesions.

In the present study, male gender, elevated serium creatine, and HbA1C levels were found to be associated with positive remodeling. Previous studies have proposed that smoking, insulin-dependent diabetes, and hyperlipidemia promote negative remodeling [28]. Furthermore, remodeling patterns may also be affected by focal lesion geometrical or hemodynamic factors [29, 30]. Negative remodeling could occur in early or advanced stage of the atherosclerotic process [31, 32]. These observations suggest that the underline mechanism of coronary arterial remodeling is complex and requires further investigations.

Positive remodeling has been thought to be a compensatory response to prevent lumen loss caused by plaque formation [26, 29], and such a process is related with vulnerable plaques and present more often in unstable clinical settings [12]. In acute coronary syndrome patients, positive remodeling lesions were associated with significantly higher revascularization rates compared with negative remodeling and intermediate remodeling lesions [33]. The VIVA study indicated that lesions resulting in MACE were associated with a higher remodeling index [20]. Our results showed that positive remodeling was closely related to greater plaque burden and echo-attenuated plaques, which is concordant with previous findings [12, 14]. However, we did not find a significant increase of risks for VOCE in the positive remodeling group. We speculate that this discrepancy may be due to several reasons. First, our study population was different from that in previous IVUS studies as we included only stable coronary artery disease patients. Second, contemporary medications which are focusing on the plaque modification and thrombosis prevention could substantially reduce the risk of larger plaque burden related to the positive remodeling lesion. Third, IVUS-derived vulnerable characteristics are still controversial; therefore, the association between positive remodeling and vulnerable plaque proposed by previous studies could be overrated.

The major finding of our study is that negative remodeling of intermediate coronary lesions was associated with more VOCE and TVR compared to intermediate and positive remodeling lesions. The explanation of this phenomenon is more likely multifactorial. The quantitative measurements of current IVUS study were consistent with previous findings which indicated that lesions with negative remodeling were associated with smaller lumen area and more severe area stenosis [34, 35]. Although negative remodeling is related with lesser plaque burden, it may, at the same time, derive little benefits from the contemporary plaque-focused treatments. Statin treatment that is frequently used in our patients could induce reverse remodeling, which seems to be a contradictory effect in the case of preexisted negative remodeling lesions [36]. In contrast, an optical coherence tomography study demonstrated that drug-eluting balloon induced expansive remodeling and may serve as a promising interventional strategy for treating negative remodeling lesions [37].

QFR is a novel angiography-derived FFR method with excellent diagnostic performance [38]. In the current study, we firstly used QFR to assess the functional significance of the IVUS-guided deferred intermediate lesions. QFR analysis demonstrated that most of the deferred intermediated lesions after IVUS examination were functionally insignificant. Although MLA and plaque burden were significantly different among three remodeling patterns, QFR was similar. This interesting phenomenon was consistent with previous finding, which reflected the heterogenous relationship between hemodynamic and multiple geometrical factors [39]. Computational fluid studies are warranted to further explore the effects of arterial remodeling on hemodynamics.

Our study showed that a higher VOCE rate for negative remodeling lesions was mainly due to repeated revascularization. These results are concordant with the lumen restrictive effect of negative remodeling. Owing to low incidence of events and stable nature of our study population, we failed to establish an association between myocardial infarction and distinct remodeling pattern.

We recognize that there are several limitations in our study. First, the study is cross-sectional for the point of coronary arterial remodeling investigation, and preoperative ischemia evaluation was not routinely performed, thereby allowing us to detect association, not to formulate causal link. Second, IVUS analysis was performed at single time point with remodeling assessment of intermediate lesions only; therefore, the conclusion of current study should be cautious to draw on the mild stenosis lesions. Finally, although we calculated the QFR to assess the functional significance of the deferred intermediate lesions, fractional flow reserve (FFR) was not evaluated in the study population. The feasibility and accuracy of QFR calculation was limited due to the retrospective nature of the current study.

## 5. Conclusion

This study indicates that negative remodeling of intermediate coronary lesions is associated with adverse long-term clinical outcomes. Novel information as such should provide important insight into the management of patients with stable coronary artery disease.

## Figures and Tables

**Table 1:** Baseline characteristics of the study group.

	IR (*n* = 72)	NR (*n* = 77)	PR (*n* = 63)	*P* value
Age, years	63.68 ± 10.93	65.43 ± 8.94	62.67 ± 8.94	0.238
Male, *n* (%)	42 (58.33)	38 (49.35)	45 (71.43)	0.030
Hypertension, *n* (%)	47 (65.28)	54 (70.13)	35 (55.56)	0.196
Hypercholesterolemia, *n* (%)	12 (16.67)	10 (12.99)	9 (14.29)	0.814
Diabetes, *n* (%)	23 (31.94)	22 (28.57)	24 (38.10)	0.484
Current smoker, *n* (%)	27 (37.50)	19 (24.68)	27 (42.86)	0.063
Prior PCI history, *n* (%)	8 (11.11)	11 (14.29)	7 (11.11)	0.795
Prior CABG history, *n* (%)	1 (1.39)	2 (2.60)	1 (1.59)	0.845
Left ventricular eject fraction (%)	62.10 ± 3.11	62.28 ± 2.65	61.66 ± 3.44	0.536
Prior MI	2 (2.78)	1 (1.30)	1 (1.59)	0.785
BMI	24.94 ± 1.32	24.65 ± 1.26	25.19 ± 1.47	0.071
Multivessel disease, *n* (%)	49 (63.64)	39 (54.17)	38 (60.32)	0.493
Laboratory				
Creatinine (*μ*mol/l)	67.17 ± 16.65	72.49 ± 17.48	76.96 ± 21.31	0.015
LDL-C (mmol/l)	2.72 ± 0.96	2.71 ± 0.93	2.54 ± 1.03	0.560
TG (mmol/l)	1.64 ± 1.13	1.60 ± 0.92	1.51 ± 0.94	0.786
HBA1c (%)	6.28 ± 1.04	6.18 ± 0.84	6.69 ± 1.36	0.044
Medications at discharge				
Aspirin, *n* (%)	58 (80.56)	54 (70.13)	46 (73.02)	0.327
Clopidogrel, *n* (%)	19 (26.39)	30 (38.96)	23 (36.51)	0.237
Statin, *n* (%)	63 (87.50)	70 (90.91)	54 (85.71)	0.621
*β*-Blocker, *n* (%)	41 (56.94)	36 (46.75)	34 (53.97)	0.440
ACEI/ARB, *n* (%)	26 (36.11)	28 (36.36)	17 (26.98)	0.426
Insulin, *n* (%)	3 (4.17)	1 (1.30)	5 (7.94)	0.153
Oral hypoglycemic agents, *n* (%)	11 (15.28)	11 (14.29)	17 (26.98)	0.109

ACEI, angiotensin-converting enzyme; ARB, angiotensin II receptor blocker; CABG, coronary artery bypass graft; HBA1c, hemoglobin A1c; IR, intermediate remodeling; LDL-C, low-density lipoprotein cholesterol; MI, myocardial infarction; NR, negative remodeling; PCI, percutaneous coronary intervention; PR, positive remodeling; TG, triglyceride.

**Table 2:** IVUS measurements.

	Remodeling of intermediate lesions			*P* value		
	IR (*n* = 72)	NR (*n* = 77)	PR (*n* = 63)	NR vs. IR	NR vs. PR	IR vs. PR
Vessel						
LAD, *n* (%)	51 (70.8)	55 (71.4)	43 (68.3)	0.94	0.68	0.75
LCX, *n* (%)	7 (9.7)	9 (11.7)	5 (7.9)	0.7	0.46	0.72
RCA, *n* (%)	14 (19.4)	13 (16.9)	15 (23.8)	0.69	0.31	0.54
Morphological analysis						
Soft, *n* (%)	12 (16.7)	13 (16.9)	22 (34.9)	0.97	0.01	0.01
Fibrous, *n* (%)	30 (41.67)	24 (31.2)	13 (20.63)	0.18	0.16	0.01
Calcified, *n* (%)	2 (2.78)	5 (6.49)	4 (6.35)	0.28	0.97	0.32
Mixed, *n* (%)	28 (38.9)	35 (45.45)	24 (38.1)	0.42	0.38	0.92
Attenuated plaque, *n* (%)	7 (9.72)	11 (14.29)	20 (31.75)	0.39	0.01	<0.01
Plaque rupture, *n* (%)	1 (1.39)	0 (0)	1 (1.59)	0.3	0.27	0.92
Lesion length	16.73 ± 6.79	17.00 ± 6.33	19.23 ± 7.56	0.8	0.05	0.04
Remodeling index	0.94 ± 0.04	0.76 ± 0.09	1.15 ± 0.12	<0.01	<0.01	<0.01
Minimal lumen site						
EEM CSA (mm^2^)	15.27 ± 4.46	11.54 ± 2.92	17.35 ± 6.20	<0.01	<0.01	0.025
Lumen CSA (mm^2^)	5.05 ± 1.39	4.16 ± 1.03	4.85 ± 1.76	<0.01	<0.01	0.48
Area stenosis (%)	54.61 ± 9.09	59.32 ± 10.15	51.67 ± 12.96	<0.01	<0.01	0.13
Plaque burden (%)	66.12 ± 6.82	63.45 ± 6.13	71.17 ± 6.45	0.01	<0.01	<0.01
Eccentricity	0.84 ± 0.13	0.79 ± 0.17	0.82 ± 0.16	0.04	0.18	0.53
Reference site						
EEM CSA at proximal reference (mm^2^)	17.84 ± 5.43	17.03 ± 5.04	16.81 ± 5.93	0.34	0.82	0.3
EEM CSA at distal reference (mm^2^)	14.83 ± 4.97	13.36 ± 3.90	13.19 ± 5.17	0.046	0.84	0.07
Lumen CSA at proximal reference (mm^2^)	12.22 ± 3.68	11.69 ± 3.91	11.32 ± 4.36	0.4	0.6	0.2
Lumen CSA at distal reference (mm^2^)	10.39 ± 3.35	9.60 ± 2.87	9.59 ± 3.22	0.13	0.97	0.16

CSA, cross-sectional area; EEM, external elastic membrane; IR, intermediate remodeling; NR, negative remodeling; PR, positive remodeling.

**Table 3:** Clinical outcomes.

	Remodeling of intermediate lesions			*P* value		
	IR (*n* = 72)	NR (*n* = 77)	PR (*n* = 63)	NR vs. IR	NR vs. PR	IR vs. PR
Cardiac death, *n* (%)	0 (0)	1 (1.3)	0 (0)	0.33	0.36	NA
TVR, *n* (%)	1 (1.4)	10 (13.0)	3 (4.8)	0.007	0.095	0.25
TVMI, *n* (%)	0 (0)	1 (1.3)	0 (0)	0.33	0.36	NA
VOCE, *n* (%)	1 (1.4)	11 (14.3)	3 (4.8)	0.004	0.06	0.25

IR, intermediate remodeling; NR, negative remodeling; PR, positive remodeling; TVR, target vessel revascularization; TVMI, target vessel-related myocardial infarction; VOCE, vessel-oriented clinical events.

**Table 4:** Independent predictors for VOCE.

	Hazard ratio	95% CI	*P* value
RI < 0.88	4.849	1.542–15.251	0.007
Previous PCI	3.309	1.128–9.703	0.029

VOCE, vessel-oriented clinical events; PCI, percutaneous coronary intervention; RI, remodeling index.

## Data Availability

The intravascular ultrasound data used to support the findings of this study are available from the corresponding author upon reasonable request.
