# Supplementary material for: Impact of Arterial Remodeling of Intermediate Coronary Lesions on Long-Term Clinical Outcomes in Patients with Stable Coronary Artery Disease: An Intravascular Ultrasound Study
Source: J Interv Cardiol. 2021 Jun 11;2021:9915759. doi: 10.1155/2021/9915759 (PMC8213497; doi:10.1155/2021/9915759)
Supplement: Supplementary Materials — The STROBE Checklist is provided in the supplementary material. [file 9915759.f1.pdf]

STROBE Statement—checklist of items that should be included in reports of observational studies

Section/item	Item No	Recommendation	Reported on Page Number/Line Number	Reported on Section/Paragraph
**Title and abstract**	1	(a) Indicate the study's design with a commonly used term in the title or the abstract	Page 2	Method
		(b) Provide in the abstract an informative and balanced summary of what was done and what was found	Page 2	Result and conlusion
**Introduction**				
Background/ rationale	2	Explain the scientific background and rationale for the investigation being reported	Page 3	Introduction
Objectives	3	State specific objectives, including any prespecified hypotheses	Page 3	Introduction
**Methods**				
Study design	4	Present key elements of study design early in the paper	Page 4	Paragraph 1
Setting	5	Describe the setting, locations, and relevant dates, including periods of recruitment, exposure, follow-up, and data collection	Page 4	Paragraph 1
Participants	6	(a) ***Cohort study***—Give the eligibility criteria, and the sources and methods of selection of participants. Describe methods of follow-up<br>***Case-control study***—Give the eligibility criteria, and the sources and methods of case ascertainment and control selection. Give the rationale for the choice of cases and controls<br>***Cross-sectional study***—Give the eligibility criteria, and the sources and methods of selection of participants	Page 4 source and inclusion criteria<br>Page 7 methods of follow-up	Paragraph 1<br>Paragraph 2
		(b) ***Cohort study***—For matched studies, give matching criteria and number of exposed and unexposed<br>***Case-control study***—For matched studies, give matching criteria and the number of controls per case	NA	
Variables	7	Clearly define all outcomes, exposures, predictors, potential confounders, and effect modifiers. Give diagnostic criteria, if applicable	Page 6	Paragraph 4
Data sources/ measurement	8*	For each variable of interest, give sources of data and details of methods of assessment (measurement). Describe comparability of assessment methods if there is more than one group	Page 5-Page 6	Paragraph1-2
Bias	9	Describe any efforts to address potential sources of bias	Page7	Paragraph 3
Study size	10	Explain how the study size was arrived at	NA	
Quantitative variables	11	Explain how quantitative variables were handled in the analyses. If applicable, describe which groupings were chosen and why	Page6	Paragraph 1

Statistical methods	12	(a) Describe all statistical methods, including those used to control for confounding	Page7	Paragraph 3
		(b) Describe any methods used to examine subgroups and interactions	NA	
		(c) Explain how missing data were addressed	No missing data	
		(d) ***Cohort study***—If applicable, explain how loss to follow-up was addressed<br>***Case-control study***—If applicable, explain how matching of cases and controls was addressed<br>***Cross-sectional study***—If applicable, describe analytical methods taking account of sampling strategy	Page8	Paragraph4
		(e) Describe any sensitivity analyses	NA	
**Results**				
Participants	13*	(a) Report numbers of individuals at each stage of study—eg numbers potentially eligible, examined for eligibility, confirmed eligible, included in the study, completing follow-up, and analysed	Figure1 flow diagram	
		(b) Give reasons for non-participation at each stage	Figure1 flow diagram	
		(c) Consider use of a flow diagram	Figure1 flow diagram	
Descriptive data	14*	(a) Give characteristics of study participants (eg demographic, clinical, social) and information on exposures and potential confounders	Page8	Paragraph1
		(b) Indicate number of participants with missing data for each variable of interest	Page8	Paragraph3
		(c) ***Cohort study***—Summarise follow-up time (eg, average and total amount)	Page8	Paragraph4
Outcome data	15*	***Cohort study***—Report numbers of outcome events or summary measures over time	Page9	paragraph1
		Case-control study—Report numbers in each exposure category, or summary measures of exposure	NA	
		Cross-sectional study—Report numbers of outcome events or summary measures	NA	
Main results	16	(a) Give unadjusted estimates and, if applicable, confounder-adjusted estimates and their precision (eg, 95% confidence interval). Make clear which confounders were adjusted for and why they were included	Page9<br>The inclusion criterion of variable for multivariable	paragraph2
		(b) Report category boundaries when continuous variables were categorized	Page9	paragraph2
		(c) If relevant, consider translating estimates of relative risk into absolute risk for a meaningful time period	Page9	paragraph2
Other analyses	17	Report other analyses done—eg analyses of subgroups and interactions, and sensitivity analyses	Page 8	Paragraph3
**Discussion**				
Key results	18	Summarise key results with reference to study objectives	Page9	Paragraph3
Limitations	19	Discuss limitations of the study, taking into account sources of potential bias or imprecision. Discuss both direction and magnitude of any potential bias	Page13	Paragraph1

Interpretation	20	Give a cautious overall interpretation of results considering objectives, limitations, multiplicity of analyses, results from similar studies, and other relevant evidence	Page10-12	
Generalisability	21	Discuss the generalisability (external validity) of the study results	Page13	Paragraph1
**Other information**				
Funding	22	Give the source of funding and the role of the funders for the present study and, if applicable, for the original study on which the present article is based	Page13	Funding statement

*Give information separately for cases and controls in case-control studies and, if applicable, for exposed and unexposed groups in cohort and cross-sectional studies.

Note: An Explanation and Elaboration article discusses each checklist item and gives methodological background and published examples of transparent reporting. The STROBE checklist is best used in conjunction with this article (freely available on the Web sites of PLoS Medicine at http://www.plosmedicine.org/, Annals of Internal Medicine at http://www.annals.org/, and Epidemiology at http://www.epidem.com/). Information on the STROBE Initiative is available at www.strobe-statement.org.

Please leave this space alone as it will be supplemented by the editorial office when needed.
